# Comparative efficacy and safety of rituximab versus cyclophosphamide with steroids in primary membranous nephropathy: a systematic review and meta-analysis

**DOI:** 10.1186/s12882-026-04895-0

**Published:** 2026-03-16

**Authors:** Fangjiao Huang, Chenxin Fu, Maorong Liu, Peiyu Wu, Yanfang Li, Zhuguang Lu, Yanlei Li, Yifeng Xie

**Affiliations:** 1 Liuzhou Hospital of Traditional Chinese Medicine Affiliated with Guangxi University of Chinese Medicine, Liuzhou, Guangxi 545001 People’s Republic of China; 2 Department of Nephrology, Liuzhou Hospital of Traditional Chinese Medicine Affiliated with Guangxi University of Chinese Medicine, Liuzhou, Guangxi 545001 People’s Republic of China

**Keywords:** Membranous nephropathy, Primary membranous nephropathy, Idiopathic membranous nephropathy, Rituximab, Cyclophosphamide, Systematic review, Meta-analysis, Treatment

## Abstract

**Background:**

Primary membranous nephropathy (PMN) is a leading cause of nephrotic syndrome in adults. In some patients, PMN can be severe or continue to worsen even after 6 months of supportive treatment. Rituximab and cyclophosphamide with steroids are used to treat moderate-risk or high-risk PMN patients, but the comparative efficacy and safety of rituximab and cyclophosphamide with steroids in patients with PMN remains debated.

**Methods:**

We searched PubMed, Embase and the Cochrane Library for the time period through November 2025 to identify randomized controlled trials and cohort studies that evaluated rituximab compared with cyclophosphamide for the treatment of PMN. The primary outcomes were complete remission (CR), partial remission (PR), and total remission (TR = PR+CR). The secondary outcomes included the relapse rate, adverse events (AEs), and serious adverse events (SAEs). The random effects model (DerSimonian–Laird method) was used to estimate relative risks (RRs) with 95% confidence intervals (CIs).

**Results:**

Eleven studies (*N* = 1068) were eligible for final inclusion. Rituximab showed similar efficacy to cyclophosphamide with steroids in inducing complete or partial remission at 6 months (RR, 0.82; 95% CI, 0.60–1.10; *p* = 0.19; *I*^2^ = 0%) and 12 months (RR, 0.85; 95% CI, 0.66–1.10; *p* = 0.21; *I*^2^ = 0%). Similarly, rates of CR were comparable between the two treatment groups at 6 months (RR, 0.97; 95% CI, 0.33–2.89; *p* = 0.95; *I*^2^ = 0%) and 12 months (RR, 0.54; 95% CI, 0.26–1.12; *p* = 0.10; *I*^2^ = 0%). There were no significant differences in the incidence of AEs or SAEs between RTX and cyclophosphamide with steroids. The RRs were 0.76 (95% CI, 0.46–1.28; *p* = 0.30; *I*^2^ = 51%) and 1.00 (95% CI, 0.50–2.00; *p* = 1.00; *I*^2^ = 0%), respectively.

**Conclusions:**

The current limited evidence suggests that RTX treatment has comparable efficacy and safety to cyclophosphamide with steroids in PMN patients at moderate risk or high risk. Further large-scale, longer follow-up, well-designed RCTs on head-to-head comparisons of the safety and efficacy of rituximab versus cyclophosphamide with steroids are urgently needed.

**Supplementary Information:**

The online version contains supplementary material available at 10.1186/s12882-026-04895-0.

## Introduction

Membranous nephropathy is an immune-mediated disease that results from the deposition of IgG and complement components onto the subepithelial layer of the glomerular capillary wall [1]. Membranous nephropathy occurs in all regions and ethnicities [2]. The annual incidence rates of membranous nephropathy are estimated at 10–12 per million in North America and 2–17 per million in Europe [3–7]. Primary membranous nephropathy (PMN) remains the most common cause of primary nephrotic syndrome in adults [8]. Previous studies reported that untreated PMN patients had spontaneous complete remission rates ranging from 20% to 30% and 10-year renal survival rates ranging from 60 to 80% [2, 8–11]. However, 30–40% of patients progress toward end-stage renal failure within 5–15 years [12].

Treatments for PMN are based on risk assessment of the progressive loss of kidney function. For patients with membranous nephropathy and at least one risk factor for disease progression, KDIGO (2021) recommends the use of rituximab or cyclophosphamide and alternate month glucocorticoids for 6 months or CNI-based therapy for ≥6 months, with the choice of treatment depending on the risk estimate [13]. Although rituximab and cyclophosphamide with steroids are used to treat moderate-risk or high-risk PMN patients, the comparative efficacy and safety of rituximab and cyclophosphamide with steroids in patients with PMN remains debated.

We performed a head-to-head comparison to assess the benefits and risks of the rituximab and cyclophosphamide with steroids protocols in patients with PMN.

## Methods

The present systematic review and meta-analysis was conducted according to the PRISMA statement [14].

### Search strategy

We searched PubMed, Embase, and the Cochrane Library up to November 2025 to identify randomized controlled trials and cohort studies that evaluated rituximab compared with cyclophosphamide with steroids for the treatment of PMN. The detailed search strategy is available in the Supplementary Materials.

### Inclusion and exclusion criteria

Studies were considered acceptable for inclusion in the meta-analysis if they met the following criteria: (1) adult PMN patients (age older than 18 years); (2) RCT or cohort studies; (3) rituximab treatment compared with cyclophosphamide plus steroids as a control; and (4) 1 or more of the following clinical outcomes reported: complete or partial remission, relapse, adverse events, and serious adverse events.

Trials were excluded if (1) they were abstracts, letters, or meeting proceedings; (2) they had repeated data or did not report outcomes of interest; or (3) the intervention was rituximab combined with another immunosuppressant (e.g., tacrolimus); or (4) the comparator was not cyclophosphamide with steroids (e.g., placebo, supportive therapy, or calcineurin inhibitors).

### Data extraction and outcome measures

Two authors (F.-J.H. and C.-X.F.) independently extracted the following data: first author, year of publication, country of author, number of patients, study design/Jadad score or study design/NOS score, rituximab group (dosage, route, and duration), cyclophosphamide group (control group: dosage, route, and duration), site (single-center or multicenter), follow-up, primary outcomes, secondary outcomes, and definition of outcomes. The primary outcomes were complete remission (CR), partial remission (PR), and total remission (TR = PR+CR). The secondary outcomes included the relapse rate, adverse events, and serious adverse events. The extracted data were entered into a standardized Excel file.

### Quality assessment

Two authors (Yifeng Xie and Fangjiao Huang) independently assessed the risk of bias of the included studies via a validated Jadad 5-point scale [15] and the quality of cohort studies via the NOA score [16]. Any disagreements were resolved by discussion and consensus.

The methodological quality of each RCT was evaluated via the Jadad scale [15]. The quality scale ranges from 0 to 5 points. Higher scores indicate better reporting. The studies are said to be of low quality if the Jadad score is ≤ 2 and high quality if the score is ≥ 3 [17].

The Newcastle‒Ottawa Scale (NOS) was employed in this meta-analysis to assess the quality of cohort studies [16]. The scale consists of 3 items describing selection (0–4 points), comparability (0–2 points), and outcome (0–3 points) in the report of a cohort study. Scores of 7–9, 4–6, and < 4 were classified as having a low, moderate, or high risk of bias, respectively.

### Statistical analysis

We estimated the relative risks (RRs) with 95% confidence interval (CI) for dichotomous outcomes. Heterogeneity of treatment effects across studies was tested using the I^2^ statistic [18, 19]. Heterogeneity was suggested if the *P*-value was ≤0.10. Statistical heterogeneity was interpreted as negligible, low, moderate, or high for *I*² values of 0–24.9%, 25–49.9%, 50–74.9%, and 75–100%, respectively [18, 19]. We pooled effect estimates via a random-effects model [20] (Mantel–Haenszel method), taking clinical heterogeneity between studies into account. To examine the influence of various factors on the treatment effects of rituximab therapy for PMN patients, we performed post hoc subgroup analyses according to baseline urinary protein levels (proteinuria > 8 g/24 h versus proteinuria < 8 g/24 h), anti-PLA2R levels (Anti-PLA2R > 80 RU/mL versus Anti-PLA2R < 80 RU/mL), eGFRs > 60 mL/min/1.73 m^2^ versus eGFRs < 60 mL/min/1.73 m^2^), sample size (*n* > 100 for large sample studies versus *n* < 100 for small sample studies), and risk of bias (low quality versus high quality). Publication bias was assessed visually via funnel plots. We performed all the statistical analyses via RevMan 5.3 (Nordic Cochrane Centre). Results were considered as statistically significant for *P* < 0.05.

## Results

### Study identification and selection

**Fig. 1:**
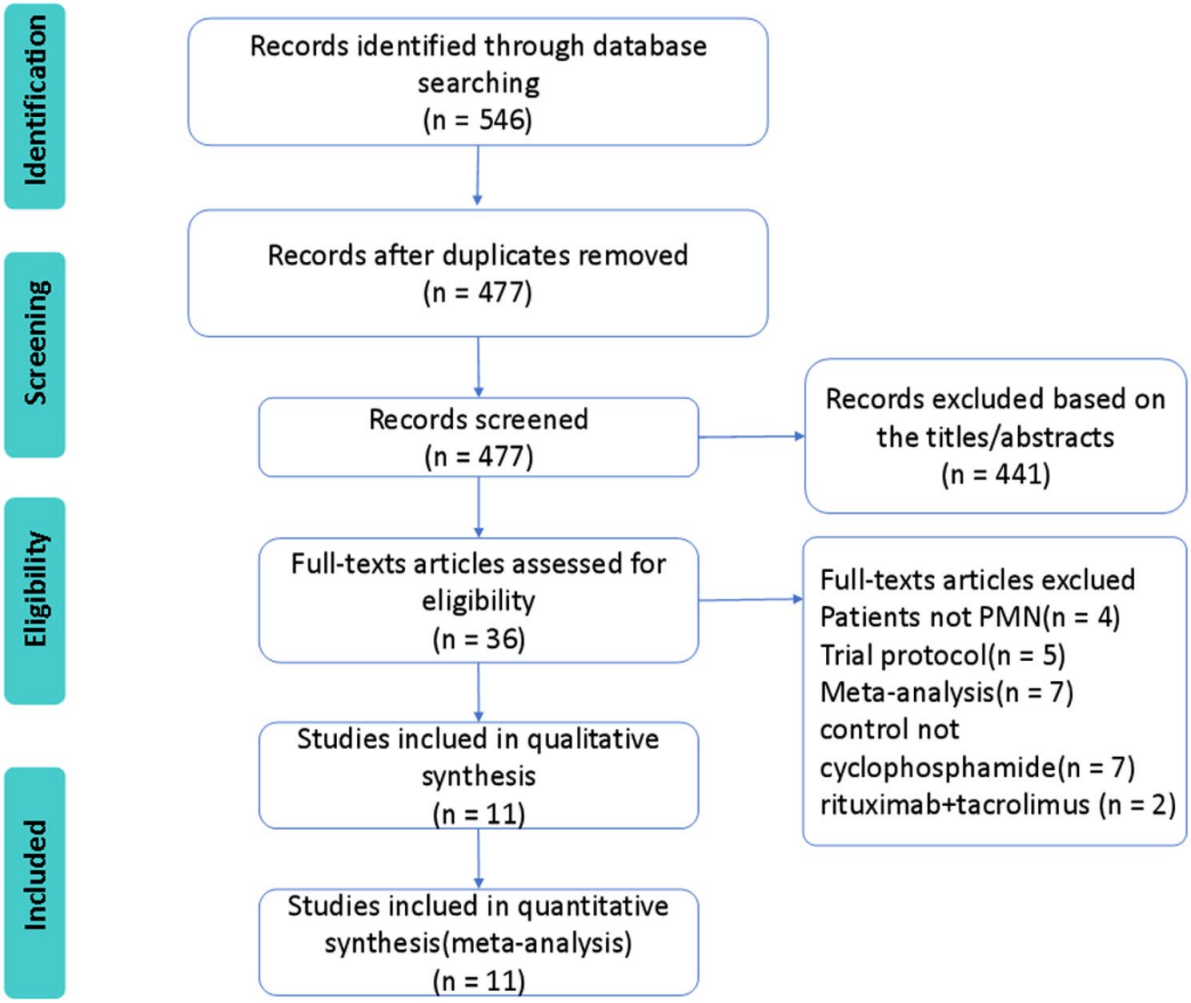
PRISMA flow diagram for study selection

The PRISMA flowchart shows the process of study identification, screening, selection, and reasons for exclusion, as shown in Fig. 1. The combined search identified a total of 477 records, 441 of which were excluded after removing duplicates and screening the titles and abstracts. A full-text assessment of 36 potentially eligible articles identified 13 studies. Two studies were excluded because their treatment was rituximab plus tacrolimus [21, 22]. Finally, 2 RCTs [23, 24] and 9 cohort studies [25–33] were included in the meta-analysis.

### Characteristics of the studies

The main characteristics of the 2 RCTs and 9 cohort studies included in the meta-analysis are presented in Table 1 (see appendix), and the outcome data of each included trial or study are described in Table 2 (see appendix). The sample size ranged from 35 to 203, with a total of 1063 patients. Only four studies [27, 28, 31, 33] had more than 100 participants. The total rituximab dose varied, including 0.375 g [30], 1 g [24], 1.5 g [26–31, 33], 2 g [23, 26, 29, 32, 33], or 3 g [25]. Cyclophosphamide treatment is cyclical [23, 24, 30] or continuous [25–28, 31–33].

Definitions of CR and PR varied across the included studies. For CR, the most common criterion was a reduction in proteinuria to ≤ 0.3 g/24h [23, 24, 26, 28–33]. The required additional criteria differed: three studies required only this proteinuria threshold [23, 30]; three also mandated stable renal function [26, 28, 33]; and four further required a serum albumin level ≥ 35 g/L [24, 29, 31, 32]. One study used a distinct definition based on normal renal function plus serum albumin > 30 g/L [25].

Definitions of PR were also heterogeneous. The most frequent component was a > 50% reduction in proteinuria to a value < 3.5 g/24 h, used by five studies [26, 28, 30, 32, 33]. Of these, four also required stable renal function [26, 28, 32, 33]. Two studies defined PR based on normal serum albumin (≥ 3.5 g/dL) plus stable creatinine [29, 31], while one study used stable renal function plus serum albumin > 30 g/L [25].

One study [27] defined partial remission as a UPCR (urinary protein‒creatinine ratio) of 0.3–3.0 g/g with at least a 50% reduction from baseline, a serum albumin concentration ≥30 g/l and stable renal function, whereas complete remission was defined as a UPCR < 0.3 g/g, a normal serum albumin concentration and stable renal function. Eight studies [23–28, 30, 31] reported relapse rate data. All studies reported AEs, and four studies [23, 24, 31, 33] reported SAEs while one study [28] provided severe adverse events.

#### Quality of the included studies

The details of the risk of bias are listed in Tables 3 and 4 (see the appendix). Overall, two RCTs [23, 24] and one cohort study [31] were categorized as having high methodological quality. Eight cohort studies [25–30, 32, 33] were categorized as having moderate methodological quality.

#### Primary outcome: composite remission (complete or partial remission) and complete remission

**Fig. 2:**
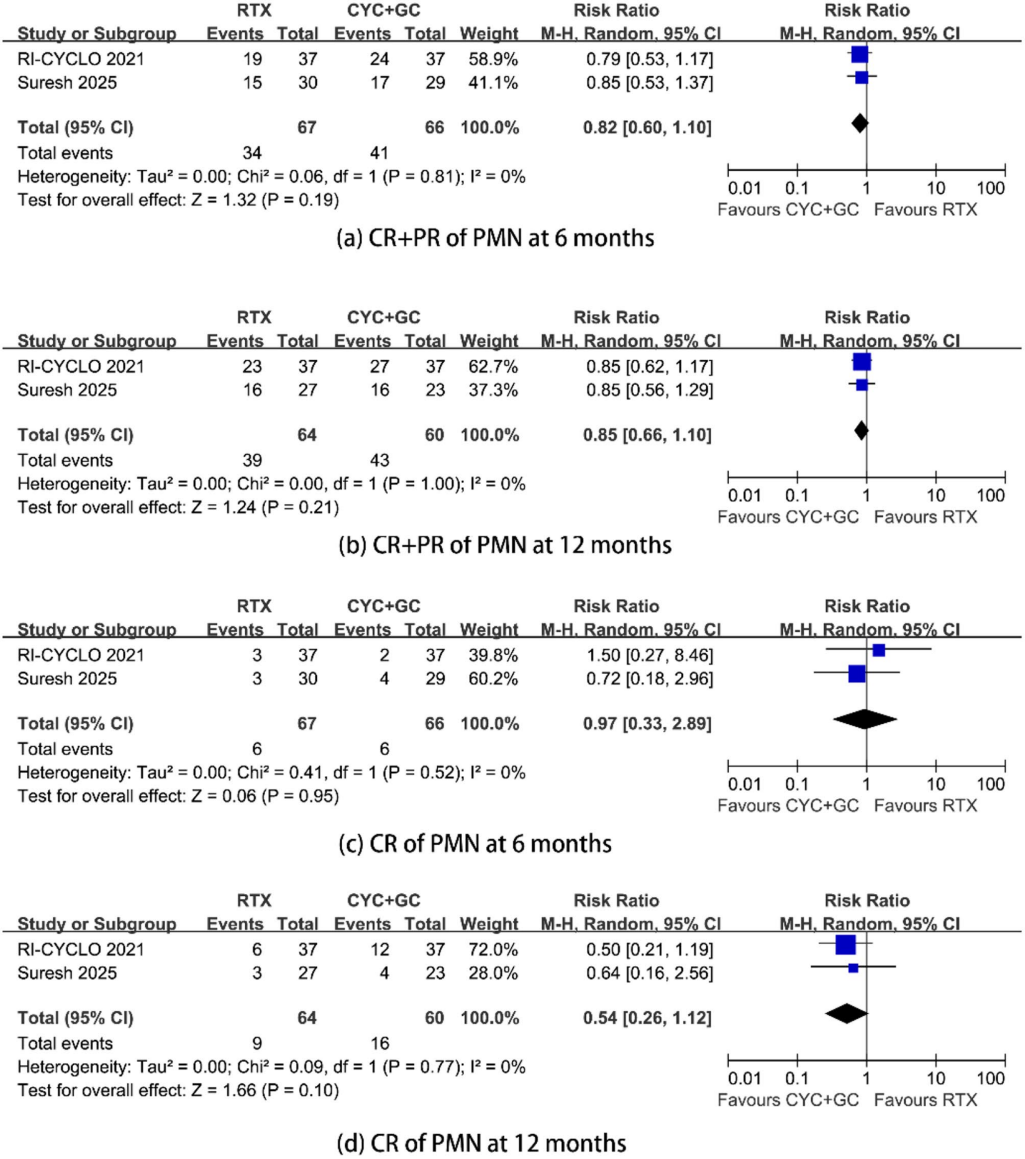
Composite remission (complete or partial) and complete remission rates in PMN patients treated with rituximab versus cyclophosphamide with steroids. (**a**) composite remission rate at 6 months, (**b**) composite remission rate at 12 months, (**c**) complete remission rate at 6 months, and (**d**) complete remission rate at 12 months

Compared with cyclophosphamide treatment, RTX treatment had similar efficacy in inducing complete or partial remission at 6 months and 12 months. The RRs were 0.82 (95% CI, 0.60–1.10; *p* = 0.19; *I*^2^ = 0%) and 0.85 (95% CI, 0.66–1.10; *p* = 0.21; *I*^2^ = 0%) (Fig. 2) (see appendix).

RTX treatment had similar efficacy, compared with cyclophosphamide treatment, in inducing complete remission at 6 months and 12 months. The RRs were 0.97 (95% CI, 0.33–2.89; *p* = 0.95; *I*^2^ = 0%) and 0.54 (95% CI, 0.26–1.12; *p* = 0.10; *I*^2^ = 0%), respectively.

#### Secondary outcome: relapse, adverse events and severe adverse events

**Fig. 3:**
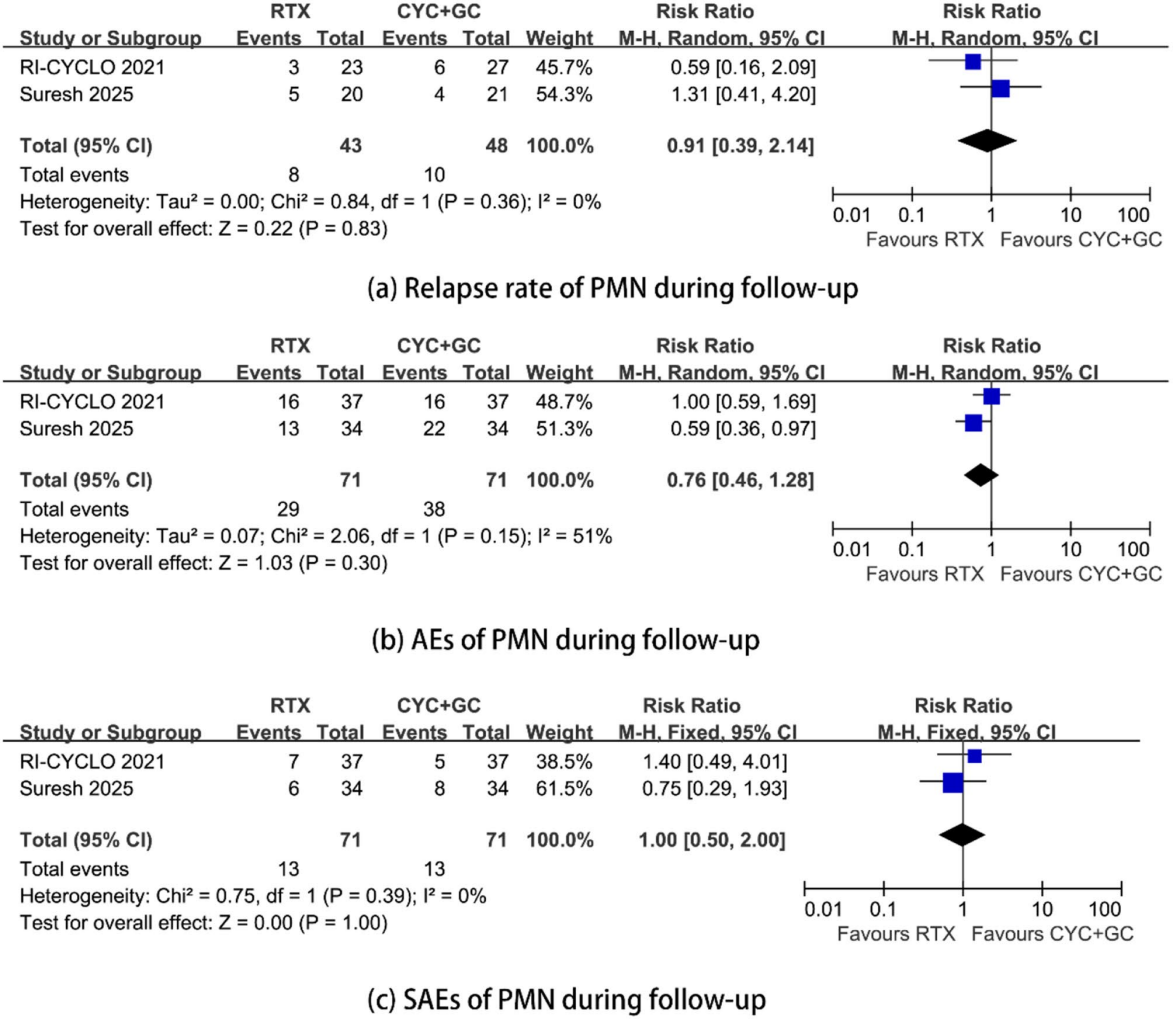
Relapse rate, incidence of adverse events and serious adverse events during follow-up in PMN patients treated with rituximab versus cyclophosphamide with steroids. (**a**) relapse rate, (**b**) incidence of adverse events, (**c**) incidence of serious adverse events

Compared with cyclophosphamide treatment, RTX treatment had a similar relapse rate during follow-up. The RR was 0.91 (95% CI, 0.39–2.14; *p* = 0.83; *I*^2^ = 0%) (Fig. 3) (see appendix).

There were no significant differences in the incidence of AEs or SAEs between the Rituximab group and the Cyclophosphamide group. The RRs were 0.76 (95% CI, 0.46–1.28; *p* = 0.30; *I*^2^ = 51%) and 1.00 (95% CI, 0.50–2.00; *p* = 1.00; *I*^2^ = 0%) (Fig. 3) (see appendix).

#### Supplementary efficacy outcomes from cohort studies

**Fig. 4:**
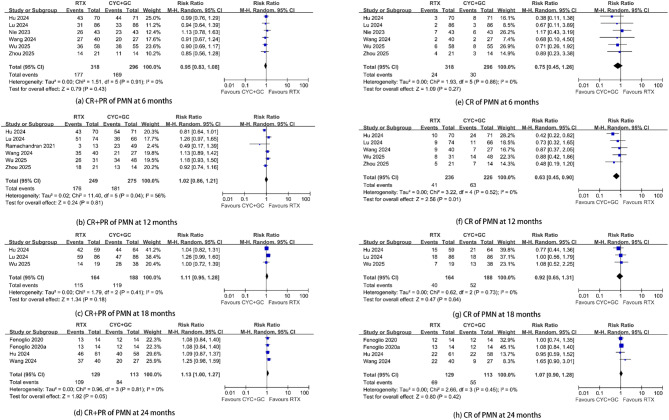
Supplementary efficacy outcomes from cohort studies. Comparison of rituximab versus cyclophosphamide plus steroids in patients with primary membranous nephropathy. (**a**–**d**) Composite remission (complete or partial) rates at 6, 12, 18, and 24 months, respectively. (**e**–**h**) Complete remission rates at the corresponding time points

Six studies [25–29, 32, 33] reported composite remission (CR + PR) at 6 months and 12 months, respectively. The pooled composite remission (CR + PR) at 6 months and 12 months was 0.95 (RR; 95% CI, 0.83–1.08; *p* = 0.43; *I*^2^ = 0%) and 1.02 (RR; 95% CI, 0.86–1.21; *p* = 0.81; *I*^2^ = 56%) (Fig. 4). Three studies [26–28, 30, 33] reported composite remission (CR + PR) at 18 months and 24 months, respectively. The pooled composite remission (CR + PR) at 18 months and 24 months was 1.11 (RR; 95% CI, 0.95–1.28; *p* = 0.18; *I*^2^ = 0%) and 1.13 (RR; 95% CI, 1.00–1.27; *p* = 0.05; *I*^2^ = 0%) (Fig. 4).

Six studies [25–29, 32, 33] and five studies [26–28, 32, 33] reported complete remission (CR) at 6 months and 12 months, respectively. The pooled complete remission (CR) at 6 months and 12 months was 0.75 (RR; 95% CI, 0.45–1.26; *p* = 0.27; *I*^2^ = 0%) and 0.63 (RR; 95% CI, 0.45–0.90; *p* = 0.001; *I*^2^ = 0%) (Fig. 4). Further exclusion of one study [28] altered the overall combined RR (CR at 12 months; RR = 0.74; 95% CI, 0.49–1.12; *p* = 0.16; *I*^2^ = 0%). Three studies [26–28, 30, 33] reported complete remission (CR) at 18 months and 24 months, respectively. The pooled complete remission (CR) at 18 months and 24 months was 0.92 (RR; 95% CI, 0.65–1.31; *p* = 0.64; *I*^2^ = 0%) and 1.07 (RR; 95% CI, 0.90–1.28; *p* = 0.42; *I*^2^ = 0%) (Fig. 4).

#### Supplementary safety outcomes from cohort studies

**Fig. 5:**
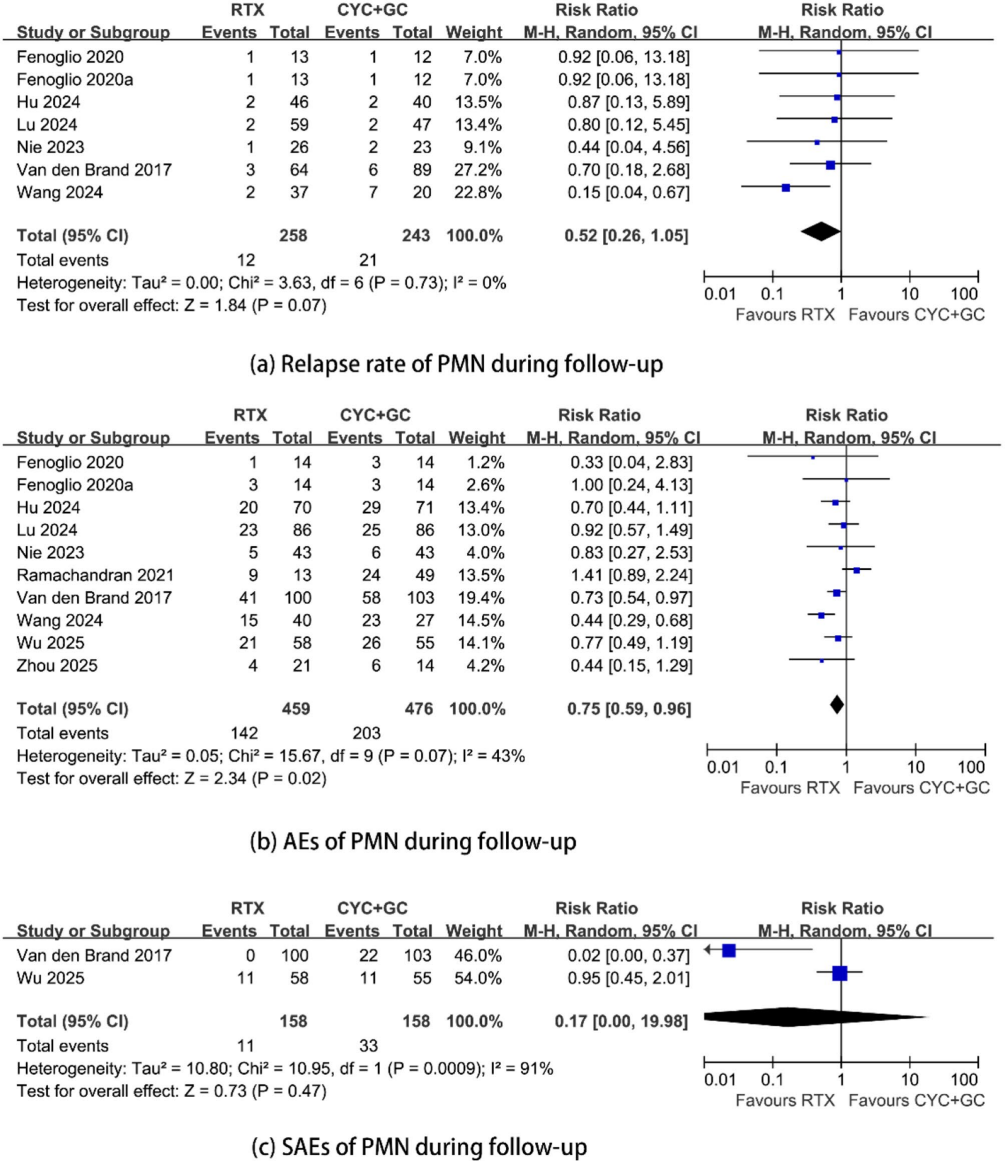
Supplementary safety outcomes from cohort studies. Comparison of rituximab versus cyclophosphamide plus steroids in patients with primary membranous nephropathy. (**a**) relapse rate; (**b**) Incidence of any adverse events; (**c**) Incidence of serious adverse events

Six studies [25–28, 30, 31] reported relapse rate during follow-up. The overall combined RR was 0.52 (95% CI, 0.26–1.05; *p* = 0.07; *I*^2^ = 0%) (Fig. 5).

All cohort studies [25–33] reported adverse events during follow-up. The overall pooled RR was 0.75 (95% CI, 0.59–0.96; *p* = 0.02; *I*^2^ = 43%) (Fig. 5). Further exclusion of any one of three studies [26, 28, 31] altered the overall pooled RR, which ranged from 0.75 (95% CI, 0.56–1.02; *p* = 0.07; *I*^2^ = 49%) to 0.82 (95% CI, 0.68–1.00; *p* = 0.05; *I*^2^ = 11%). The results of subgroup analyses are presented in Table 5.

Only two studies [31, 33] reported serious adverse events during follow-up. The overall combined RR was 0.17 (95% CI, 0.00–19.98; *p* = 0.47; *I*^2^ = 91%) (Fig. 5).

### Publication bias

Publication bias was not assessed quantitatively (e.g., via funnel plots or statistical tests) due to the limited number (< 10) of studies included in each analysis.

## Discussion

This meta-analysis indicates that rituximab has comparable efficacy to cyclophosphamide with steroids for promoting disease remission (CR + PR or CR) in patients with primary membranous nephropathy. In addition, the RTX group has similar relapse rate and incidence of adverse events and serious adverse events than did the cyclophosphamide group. These results should be cautiously interpretated because of underlying heterogeneity due to small number of our included studies and small sample size.

The Chi^2^ test has low power in the situation of a meta-analysis when studies have small sample size or are few. This means that while a statistically significant result may indicate a problem with heterogeneity, a non-significant result must not be taken as evidence of no heterogeneity in number [18, 19]. Any pooled estimates (composite remission, complete remission, relapse rate etc.) in our meta-analysis had few included studies (2 to 9 for any pooled effect, *n* < 10). Among the 11 included studies, 7 out of 11 had small sample size (*N* < 100). Therefore, results from heterogeneity tests (e.g., Chi², p-value, I²) should be interpreted cautiously.

Several sources of heterogeneity are evident across the included studies. These include: (1) variability in patient characteristics, such as baseline 24-hour urinary protein (> 8 g vs. <8 g) and anti-PLA2R antibody levels (> 80 vs. <80 RU/mL); (2) differences in treatment protocols, particularly the wide range of total rituximab doses administered (0.375 g to 3.0 g); (3) considerable variation in follow-up duration (6 to 40 months); and (4) the use of non-identical definitions for complete and partial remission across different trials. Definitions of CR and PR varied across the included studies.

Compared with recently published meta-analyses [34–39], however, this meta-analysis revealed several differences. First, cohort studies were included because membranous nephropathy is a disease with serious, objective, clinical outcomes such as mortality or kidney failure [13]. This meta-analysis uses information from cohort studies as part of the evidence base. Second, this meta-analysis showed that rituximab had comparative efficacy and safety profile to cyclophosphamide with steroids in patients with primary membranous nephropathy. Third, this meta-analysis included recently published studies and thus had a larger sample size.

This meta-analysis has several potential limitations that should be taken into account. First, the number of included RCTs was small, and the studies had small sample sizes and short follow-up periods. Second, the number of included trials was too small to evaluate publication bias in our meta-analysis, either visually via funnel plots or quantitatively via Egger’s test [40] and Begg’s test [41]. There may be potential publication bias in this meta-analysis. Third, comparisons between rituximab and cyclophosphamide are based on clinical responses (complete remission or partial remission), and clinical outcomes such as kidney failure, ESRD, and quality of life were not reported.

Further studies should focus on the following points. There is an urgent need for large-scale, adequately powered, well-designed RCTs that compare the long-term (2–5 years) efficacy and safety of rituximab treatment and cyclophosphamide treatment. Next, such RCTs should have longer follow-up periods (2–5 years) to assess comparative efficacy and safety of rituximab treatment versus cyclophosphamide with steroids in PMN. Third, such RCTs should standardize the treatment protocol (i.e., further consistency regarding dosage, route, follow-up, duration of administration, and long-term outcomes).

## Conclusions

The current limited evidence suggests that rituximab treatment has comparable efficacy and safety to cyclophosphamide with steroids in patients with primary membranous nephropathy. The results should be interpreted with caution because of the heterogeneity among study designs. Further large-scale, longer follow-up, well-designed RCTs on head-to-head comparisons of the safety and efficacy of RTX vs. cyclophosphamide with steroids are urgently needed.

## Appendix

**Table 1:** Main characteristics of the studies included in the meta-analysis

Author	Year	Country	Sample Size(n)	Study Design/Jadad or NOS Score	Site	Treatment	Regimens	Follow-up	Outcomes
Scolari [23] (RI-CYCLO)	2021	Italy & Switzerland	37	RCT/3	M	RTX	received two courses of rituximab at a dose of 1 g on days 1 and 15	36 months	CR, PR, TR at 6/9/12/18/24/36 months Relapse, AEs, SAEs
			37			CYC + GC	three consecutive cycles lasting for 2 months each (for a total of 6 months), where steroids were alternated with cyclophosphamide every other month; the cumulative dose of cyclophosphamide per patient was 180 mg/kg		
Suresh [24]	2025	India	34	RCT/3	S	RTX	RTX injection (500 mg) IV given on days 1 and 15. Each patient received premedication with injection methylprednisolone (125 mg), injection Pheniramine (45.5 mg), and tablet Paracetamol (500 mg)	12 months	CR, PR, TR at 6/12 months Relapse, AEs, SAEs
			34			CYC + GC	Cyclical therapy with 1 g IV methylprednisolone daily (Days 1–3), then oral prednisolone (0.5 mg/kg/day) for 27 days (Days 4–30) on months 1, 3, and 5, alternating with oral cyclophosphamide (2.0 mg/kg/day) for 30 days on months 2, 4, and 6.		
Van den Brand [31]	2017	Italy & Switzerland	100	RC/7	M	RTX	four weekly doses of 375 mg/m2 RTX infused intravenously.	40 months	CR, PR, TR at last follow-up, Relapse, AEs, SAEs
			103			CYC + GC	Oral cyclophosphamide (1.5 mg/kg daily) was administered for 6–12 months, and methylprednisolone pulses (1 g) were infused on days 1–3, 61–63, and 121–123 in combination with 0.5 mg/kg oral prednisone every other day for 5 months before tapering.		
Zhou [32]	2025	China	21	RC/5	S	RTX	1000 mg on day 1 and day 15	12 months	CR, PR, TR at 6/12 months, AEs
			14			CYC + GC	intravenous infusion (0.5 to 0.75 g/m2/month, maximum dose is 1 g/month, the cumulative dose of cyclophosphamide − 9 to 10 g		
Ramachandran [29]	2021	India	13	PC/6	S	RTX	3 received weekly (×4) doses of 375 mg/m^2^ , 4 received 2 doses of 1 g (×2) 15 days apart, and 6 received CD-19 targeted dosing	26 months	TR at 12/24 months, AEs
			49			CYC + GC	Cyclical Regimen		
Fenoglio [30]	2020	Italy	14	RC/6	M	Low-Dose RTX	one dose of RTX 375 mg/m^2^	24 months	CR, PR, TR at 24 months, Relapse, AEs
			14			Standard RTX	four weekly doses of rituximab 375 mg/m^2^		
			14			CYC + GC	Ponticelli Regimen: patients received methylprednisolone at months 1, 3, and 5 (1 g intravenously at days 1, 2, and 3, then 0.5 mg/kg/day orally from day 4 to day 30). At months 2, 4, and 6, patients received oral cyclophosphamide adjusted for age and renal function (1.0–2.0 mg/kg/day for 30 days)		
Nie [25]	2023	China	43	RC/5	S	RTX	one dose of rituximab 375 mg/m^2^, once weekly	6 months	CR, PR, TR at 6 months, Relapse, AEs
			43			CYC + GC	0.8–1.0 g intravenously, once monthly; If clinical complete or partial remission is achieved after 6 months of treatment, continue therapy for an additional 3 months (cumulative dose < 10 g)		
Lu [27]	2024	China	86	RC/6	S	RTX	a dose of 375 mg/m^2^ intravenous rituximab once every two weeks until their peripheral blood B-cell count was 0/µl.	18 months	CR, PR, TR at 6/12/18 months, Relapse, AEs
			86			CYC + GC	a single dose of 500–750 mg/m^2^ by intravenous infusion monthly for the initial 6 months, and once every 2–3 months for the later period. All patients in CYC group received a combination of oral prednisone 0.6–0.8 mg/kg/d for 4–8 weeks, and gradually tapered.		
Wang [26]	2024	China	40	RC/6	M	RTX	received intravenous RTX at a dose of 375 mg/m^2^ once a week for 4 consecutive weeks or 1 g every 2 weeks (a total of two times as a course of treatment). At least two doses of 375 mg/m^2^ or one dose of 1 g RTX therapy was repeated 4–6 months later to maintain the effect of RTX.	24 months	CR, PR, TR at 6/12/24 months, Relapse, AEs
			27			CYC + GC	the initial oral corticosteroid dose was 1 mg/kg/day (maximum dose was 60 mg), tapered after 8–12 weeks until withdrawal. Intermittent intravenous CYC was given monthly at 10–15 mg/kg for six months (generally 0.6–1.0 g/month), followed by an additional dose of the same approximately every 3 months for the next 6 months. The total dose of CTX generally reached 6–8 g and did not exceed 10 g.		
Hu [28]	2024	China	70	PC/6	S	RTX	a dose of 375 mg/m2 every week for a total of 4 doses. Methylprednisolone 40 mg i.v. will be given before the first dose. Chlorpheniramine (10 mg) and acetaminophen (500 mg) or other similar drugs will be given orally before each infusion to prevent allergic reactions	24 months	CR, PR, TR at 6/12/18/24 months, Relapse, AEs, SAEs
			71			CYC + GC	oral prednisone starting at 0.8 mg/kg/24 h combined with intravenous CTX at a dose of 500 mg/m2 every 4 weeks until the total amount administered reached 6–8 g. Oral prednisone was maintained for 8 weeks and then tapered gradually to 5–10 mg every 4 weeks and may be stopped at 1 year		
Wu [33]	2025	China	58	RC/6	S	RTX	received either a weekly intravenous infusion of 375 mg/m² for four consecutive weeks, or 1 g every two weeks (for a total of two doses as one treatment course). To reduce infusion reactions, patients received 40 mg of intravenous methylprednisolone prior to each infusion.	18 months	CR, PR, TR at 6/12/18 months, AEs, SAEs
			55			CYC + GC	the initial oral corticosteroid dose was 1 mg·kg⁻¹·d⁻¹ (maximum 60 mg), which was gradually tapered off and discontinued after 8–12 weeks. Cyclophosphamide was administered intermittently via intravenous infusion at a monthly dose of 10–15 mg/kg for 6 months (typically 0.6–1.0 g/month), followed by additional doses administered approximately every 3 months over the next 6 months. The total cumulative dose generally reached 6–8 g and did not exceed 10 g.		

RCT: randomized controlled trial, PC: prospective cohort, RC: retrospective cohort, M: multicenter, S: single-center, RTX: rituximab, TAC: tacrolimus, CYC: cyclophosphamide, GC: glucocorticoids. CR: complete remission, a reduction in proteinuria from baseline to a value ≤ 0.3 g/24 h plus stable kidney function (eGFR > 45 ml/min per 1.73 m2); PR: partial remission, a reduction in proteinuria > 50% from baseline; and a value < 3.5 g/24 h plus stable renal function (eGFR > 45 ml/min per 1.73 m2). TR: total remission, TR = CR+PR. Relapse: a reappearance of proteinuria > 3.5 g/24 h and at least a > 50% increase from the lowest value at 3 or more consecutive visits in patients with previous partial or complete remission. AEs: adverse events, any untoward medical occurrence; SAEs: serious adverse events, any untoward medical occurrence that resulted in death, was life-threatening, required inpatient hospitalization or resulted in persistent or significant disability/incapacity

**Table 2:** Outcome data of the 11 included studies

Author	Year	Treatment	Sample Size(n)	Urinary protein (g/24 h)	CR				TR(PR + CR)				Relapse	AEs	SAE patients	Follow-up
					6 months	12 months	18 months	24 months	6 months	12 months	18 months	24 months				
Scolari [23] (RI-CYCLO)	2021	RTX	37	6.1 (4, 10.1)	3/37	6/37	10/32	11/26	19/37	23/37	21/32	22/26	3/23	16/37	7/37	24 months
		CYC + GC	37	6.2 (5.1, 9.3)	2/37	12/37	7/34	11/31	24/37	27/37	27/34	25/31	6/27	16/37	5/37	
Suresh [24]	2025	RTX	34	NA	3/30	3/27	NA	NA	15/30	16/27	NA	NA	5/20	13/34	6/34	12 months
		CYC + GC	34	NA	4/29	4/23	NA	NA	17/29	16/23	NA	NA	4/21	22/34	8/34	
Van den Brand [31]	2017	RTX	100	NA	NA	NA	NA	26/100^a^	NA	NA	NA	64/100^a^	3/64	41/100	0/100	40 months
		CYC + GC	103	NA	NA	NA	NA	34/103^a^	NA	NA	NA	89/103^a^	6/89	58/103	22/103	
Zhou [32]	2025	RTX	21	8.46(6.60,12.46)	4/21	5/21	NA	NA	14/21	18/21	NA	NA	NA	4/21	NA	12 months
		CYC + GC	14	7.80(5.41,13.73)	3/14	7/14	NA	NA	11/14	13/14	NA	NA	NA	6/14	NA	
Ramachandran [29]	2021	RTX	13	7.9 (4.5, 13.5)	NA	NA	NA	NA	NA	3/13	NA	5/13a	NA	9/13	NA	24 months
		CYC + GC	49	5.2 (4, 8)	NA	NA	NA	NA	NA	23/49	NA	24/49a	NA	24/49	NA	
Fenoglio [30]	2020	Low-dose RTX	14	7.5 ± 4.8	NA	NA	NA	12/14	NA	NA	NA	13/14	1/13	1/14	NA	24 months
		Standard RTX	14	5.1 ± 1.41	NA	NA	NA	13/14	NA	NA	NA	13/14	1/13	3/14	NA	
		CYC + GC	14	8.27 ± 4.78	NA	NA	NA	12/14	NA	NA	NA	12/14	1/12	3/14	NA	
Nie [25]	2023	RTX	43	6.02 ± 2.01	7/43	NA	NA	NA	26/43	NA	NA	NA	1/26	5/43	NA	6 months
		CYC	43	5.87 ± 1.98	6/43	NA	NA	NA	23/43	NA	NA	NA	2/23	6/43	NA	
Lu [27]	2024	RTX	86	NA	2/86	9/74	18/86	NA	31/86	51/74	59/86	NA	2/59	23/86	NA	12 months
		CYC + GC	86	NA	3/86	11/66	18/86	NA	33/86	36/66	47/86	NA	2/47	25/86	NA	
Wang [26]	2024	RTX	40	6.78 (4.68, 8.89)	2/40	9/40	NA	22/40	27/40	35/40	NA	37/40	2/37	15/40	NA	24 months
		CYC	27	6.11 (4.45, 8.43)	2/27	7/27	NA	9/27	20/27	21/27	NA	20/27	7/20	23/27	NA	
Hu [28]	2024	RTX	70	8.13 (4, 21.14)	3/70	10/70	15/59	22/61	43/70	43/70	42/59	46/61	2/46	20/70	2/70^b^	24 months
		CYC + GC	71	7.94 (4.11, 20.1)	8/71	24/71	21/64	22/58	44/71	54/71	44/64	40/58	2/40	29/71	6/71^b^	
Wu [33]	2025	RTX	58	4.58 (0.10, 15.27)	6/58	8/31	7/19	NA	36/58	26/31	14/19	NA	NA	21/58	11/58	18 months
		CYC + GC	55	4.11 (0.26, 13.13)	8/55	14/48	13/38	NA	38/55	34/48	28/38	NA	NA	26/55	11/55	

a: data at the last follow-up; b: data for severe adverse events; NA: Not applicable

**Table 3:** Assessing the risk of bias of the included studies via the validated Jadad 5-point scale

RCTs	Sample size (*n*)	Study Design	Randomization (0–2 points)	Double-blind (0–2 points)	Drop-outs and withdrawals (0–1 points)	Jadad score
RI-CYCLO 2021 [23]	74	multicenter, open-labeled, parallel	2	0	1	3
Suresh 2025 [24]	68	single-center, open-labeled, parallel	2	0	1	3

**Table 4:** Assessment of the quality of the included cohort studies by the NOS [16]

Studies	Selection				Comparability	Outcome assessment			NOS score
	1)	2)	3)	4)	5)	6)	7)	8)	
van den Brand 2017 [31]	a	a	a	a	a	b	a	b	7
Fenoglio 2021 [30]	a	a	a	a	a	b	a	d	6
Ramachandran 2021 [29]	a	a	a	a	a	b	a	d	6
Zhou 2025 [32]	a	a	a	a	a	b	b	d	5
Nie 2023 [25]	a	a	a	a	a	b	b	d	5
Lu 2024 [27]	a	a	a	a	a	b	a	d	6
Wang 2024 [26]	a	a	a	a	a	b	a	d	6
Hu 2024 [28]	a	a	a	a	a	b	a	d	6
Wu 2025 [33]	a	a	a	a	a	b	a	d	6

(1) Representativeness of the exposed cohort; (2) selection of the non-exposed cohort; (3) ascertainment of exposure; (4) demonstration that outcome of interest was not present at start of study; (5) comparability of cohorts on the basis of the design or analysis; (6) assessment of outcome; (7) was follow-up long enough for outcomes to occur; (8) adequacy of follow up of cohorts

The letters a, b, and d denote the response options for the eight items within the three domains (Selection, Comparability, and Outcome Assessment) of the Newcastle–Ottawa Scale (NOS). These options are ordered alphabetically in descending score value, where ‘a’ corresponds to the highest score and ‘d’ to the lowest

**Table 5:** Subgroup analyses of adverse events of PMN patients at last follow-up (cohort studies)

Subgroups	No. of studies	No. of patients	Relative Risk (95% CI)	*P*-value	I^2^(%)	*P*_interaction_
All studies [25–33]	9	935	0.75(0.59, 0.96)	0.02	43%	
Baseline urinary protein level						0.48
< 8 g/24 h [25–27, 29–31, 33]	7	759	0.78(0.59, 1.05)	0.10	52%	
> 8 g/24 h [28, 32]	2	176	0.65(0.43, 1.00)	0.05	0%	
Baseline anti-PLA2R level						0.85
< 80 RU/mL [27, 28, 30–32]	5	607	0.74(0.60, 0.91)	0.005	0%	
> 80 RU/mL [25, 26, 29, 33]	4	328	0.79(0.45, 1.38)	0.40	77%	
Baseline eGFRs						0.26
< 60 mL/min/1.73 m^2^ [29, 31]	2	265	0.99(0.51, 0.91)	0.98	83%	
> 60 mL/min/1.73 m^2^ [25–28, 30, 32, 33]	7	670	0.67(0.53, 0.83)	0.0003	4%	
Sample size						0.82
*n* < 100 [25, 26, 29, 30, 32]	5	306	0.71(0.39, 1.29)	0.26	67%	
*n* > 100 [27, 28, 31, 33]	4	629	0.76(0.62, 0.92)	0.006	0%	

P-value: Test for overall effect; P_interaction_: Test of interaction;

## Supplementary Information

Below is the link to the electronic supplementary material.

Supplementary Material 1

Supplementary Material 2

## Data Availability

All the data generated or analyzed during this study are included in this published article and its supplementary information files.

## Abbreviations

PMN: Primary membranous nephropathy
RTX: Rituximab
TAC: Tacrolimus
CYC: Cyclophosphamide
GC: Glucocorticoids
RCT: Randomized controlled trial
PC: Prospective cohort
RC: Retrospective cohort
M: Multicenter
S: Single-center
CI: Confidence interval
CR: Complete remission
PR: Partial remission
TR: Total remission, TR = CR + PR
AEs: Adverse events
SAEs: Serious adverse events
KDIGO: Kidney Disease: Improving Global Outcomes
PRISMA: Preferred Reporting Items for Systematic Reviews and Meta-Analyses
RR: Relative risk
ALB: Albumin
