# Supplementary material for: Comparative efficacy and safety of rituximab versus cyclophosphamide with steroids in primary membranous nephropathy: a systematic review and meta-analysis
Source: BMC Nephrol. 2026 Mar 16;27:256. doi: 10.1186/s12882-026-04895-0 (PMC13104270; doi:10.1186/s12882-026-04895-0)
Supplement: Supplementary file 2 — Supplementary Material 2 [file 12882_2026_4895_MOESM2_ESM.docx]

**Supplement table 4**. Assessment of the quality of the included studies by NOS.

| **Studies** | **Selection** | | | | **Comparability** | **Outcome assessment** | | | **NOS score** |
| --- | --- | --- | --- | --- | --- | --- | --- | --- | --- |
|  | 1) | 2) | 3) | 4) | 5) | 6) | 7) | 8) |  |
| van den Brand 2017[31] | a | a | a | a | a | b | a | b | 7 |
| Fenoglio 2021[30] | a | a | a | a | a | b | a | d | 6 |
| Ramachandran 2021[29] | a | a | a | a | a | b | a | d | 6 |
| Zhou 2025[32] | a | a | a | a | a | b | b | d | 5 |
| Nie 2023[25] | a | a | a | a | a | b | b | d | 5 |
| Lu 2024[27] | a | a | a | a | a | b | a | d | 6 |
| Wang 2024[26] | a | a | a | a | a | b | a | d | 6 |
| Hu 2024[28] | a | a | a | a | a | b | a | d | 6 |
| Wu 2025[33] | a | a | a | a | a | b | a | d | 6 |

1) Representativeness of the exposed cohort; 2) selection of the non-exposed cohort; 3) ascertainment of exposure; 4) demonstration that outcome of interest was not present at start of study; 5) comparability of cohorts on the basis of the design or analysis; 6) assessment of outcome; 7) was follow-up long enough for outcomes to occur; 8) adequacy of follow up of cohorts.

**NEWCASTLE - OTTAWA QUALITY ASSESSMENT SCALE**

**COHORT STUDIES**

Note: A study can be awarded a maximum of one star for each numbered item within the Selection and Outcome categories. A maximum of two stars can be given for Comparability

**Selection**

1) Representativeness of the exposed cohort

a) truly representative of the average _______________ (describe) in the community **¯**

b) somewhat representative of the average ______________ in the community **¯**

c) selected group of users eg nurses, volunteers

d) no description of the derivation of the cohort

2) Selection of the non exposed cohort

a) drawn from the same community as the exposed cohort **¯**

b) drawn from a different source

c) no description of the derivation of the non exposed cohort

3) Ascertainment of exposure

a) secure record (eg surgical records) **¯**

b) structured interview **¯**

c) written self report

d) no description

4) Demonstration that outcome of interest was not present at start of study

a) yes **¯**

b) no

**Comparability**

1) Comparability of cohorts on the basis of the design or analysis

a) study controls for _____________ (select the most important factor) **¯**

b) study controls for any additional factor **¯** (This criteria could be modified to indicate specific control for a second important factor.)

**Outcome**

1) Assessment of outcome

a) independent blind assessment **¯**

b) record linkage **¯**

c) self report

d) no description

2) Was follow-up long enough for outcomes to occur

a) yes (select an adequate follow up period for outcome of interest) **¯**

b) no

3) Adequacy of follow up of cohorts

a) complete follow up - all subjects accounted for **¯**

b) subjects lost to follow up unlikely to introduce bias - small number lost - > ____ % (select an adequate %) follow up, or description provided of those lost) **¯**

c) follow up rate < ____% (select an adequate %) and no description of those lost

d) no statement.
